# Identification of alternatively spliced Dab1 and Fyn isoforms in pig

**DOI:** 10.1186/1471-2202-12-17

**Published:** 2011-02-05

**Authors:** Huan Long, Hans H Bock, Ting Lei, Xuejun Chai, Jihong Yuan, Joachim Herz, Michael Frotscher, Zaiqing Yang

**Affiliations:** 1 Key Laboratory of Agricultural Animal Genetics, Breeding and Reproduction of Ministry of Education, College of Life Science and Technology, Huazhong Agricultural University, Wuhan, 430070, PR China; 2 Institute of Anatomy and Cell Biology, University of Freiburg, Freiburg, Germany; 3 Department of Medicine 2, University Hospital Freiburg, Freiburg, Germany; 4 Center for Neurosciences, University of Freiburg, Freiburg, Germany; 5 Department of Molecular Genetics, University of Texas Southwestern Medical Center, Dallas, Texas, USA

## Abstract

**Background:**

Disabled-1 (Dab1) is an adaptor protein that is essential for the intracellular transduction of Reelin signaling, which regulates the migration and differentiation of postmitotic neurons during brain development in vertebrates. Dab1 function depends on its tyrosine phosphorylation by Src family kinases, especially Fyn.

**Results:**

We have isolated alternatively spliced forms of porcine Dab1 from brain (sDab1) and liver (sDab1-Li) and Fyn from brain (sFyn-B) and spleen (sFyn-T). Radiation hybrid mapping localized porcine Dab1 (sDab1) and Fyn (sFyn) to chromosomes 6q31-35 and 1p13, respectively. Real-time quantitative RT-PCR (qRT-PCR) demonstrated that different isoforms of Dab1 and Fyn have tissue-specific expression patterns, and sDab1 and sFyn-B display similar temporal expression characteristics in the developing porcine cerebral cortex and cerebellum. Both sDab1 isoforms function as nucleocytoplasmic shuttling proteins. It was further shown that sFyn phosphorylates sDab1 at tyrosyl residues (Tyr) 185, 198/200 and 232, whereas sDab1-Li was phosphorylated at Tyr 185 and Tyr 197 (corresponding to Y232 in sDab1) in vitro.

**Conclusions:**

Alternative splicing generates natural sDab1-Li that only carries Y185 and Y197 (corresponding to Y232 in sDab1) sites, which can be phosphorylated by Fyn in vitro. sDab1-Li is an isoform that is highly expressed in peripheral organs. Both isoforms are suggested to be nucleocytoplasmic shuttling proteins. Our results imply that the short splice form sDab1-Li might regulate cellular responses to different cell signals by acting as a dominant negative form against the full length sDab1 variant and that both isoforms might serve different signaling functions in different tissues.

## Background

Disabled-1 (Dab1) was originally isolated as a cytoplasmic Src-binding protein that becomes strongly tyrosine phosphorylated when co-expressed with constitutively activated Src[1]. Binding of Reelin, a large secreted signaling protein, to the extracellular domains of VLDLR and ApoER2 rapidly induces tyrosine phosphorylation of Dab1[2-4], which is required for proper positioning of neurons in the developing mammalian brain. Moreover, Dab1 contains two leucine-rich nuclear export signal (NES) sequences and a bipartite nuclear localization signal (NLS) sequence and functions as a nucleocytoplasmic shuttling protein[5]. Efficient tyrosine phosphorylation of Dab1 requires the activation of Src family non-receptor tyrosine kinases (SFKs), and SFKs can undergo autoactivation in the presence of phosphorylated Dab1[6]. As a member of the SFKs, Fyn tyrosine kinase is a critical regulator of Dab1 during brain development[7]. *Reeler*-like phenotypes were found in mutants deficient in Dab1[8] or both Src tyrosine kinase family members Fyn and Src[9].

Phosphorylation of Dab1 is required for proper Dab1 function. There are five tyrosine residues in Dab1 (Y185, Y198, Y200, Y220 and Y232), which correspond to four conserved tyrosine phosphorylation sites. Mice with phenylalanine substitutions of all of these five tyrosines exhibit a *Reeler *phenotype[10]. It was reported that Src can phosphorylate all five tyrosines in vitro, but that Y198 and Y220 represent the major sites of Reelin-induced Dab1 phosphorylation in embryonic neurons[2]. Binding of Lis1 to Dab1 also depends on phosphorylation at either Y198 or Y220[11]. Phosphorylation at both Y220 and Y232 was shown to be important for binding to members of the Crk family of adaptor proteins[12,13]. These residues are required for the detachment of migrating neurons from radial glial fiber[14] and are also essential for Nckβ binding[15].

Functions of Dab1 and Fyn could be regulated at the transcript level by alternative splicing. Isoforms of Dab1 are species-specific and have different tissue expression profiles. Different mRNAs encode at least four mouse Dab1 isoforms of 555, 555*, 217 and 271 amino acids. In early embryonic mouse brain, the Dab1 isoform 555* was predominant, while in later stages Dab1 does not contain this fragment[1,16]. In human, there are two Dab1 isoforms, 555 and 555*[16]. Similarly, at least three alternatively spliced forms of Dab1 are expressed at different stages during zebrafish development[17]. Two alternatively spliced variants of Dab1 were isolated from chick retina: an early form (chDab1-E) expressed in undifferentiated cells, and a late form (chDab1-L) expressed in amacrine and ganglion cells[18]. Two distinct Fyn isoforms exhibit mutually exclusive expression patterns; one is found mainly in the brain (Fyn-B), while the second form is found in the immune system, e.g. in thymocytes and splenocytes (Fyn-T)[19]. A third splice-variant of Fyn (FynΔ7), in which exon 7 is absent, was isolated from peripheral blood mononuclear cells[20].

Pig is an important animal model for genetic and biomedical research because of the similarities between human and swine anatomy, biochemistry, physiology, size and genetics[21]. Pig models for the study of the developing brain[22,23], retinal disease[24] and pluripotent stem cells[25] have been developed. Recently, the structure and developmental expression of pig Reelin was described [26]. In the present study, alternatively spliced isoforms of *Dab1 *and *Fyn *were isolated from pig, and the different isoforms exhibited tissue-specific expression patterns. Moreover, we studied the chromosome localization, subcellular distribution and interaction of Dab1 and Fyn. These data provide basic molecular information useful for further studies on the function of Dab1 and Fyn isoforms in the pig and support the usefulness of this animal model in the study of neurodevelopmental disorders.

## Results

### Cloning of porcine *Dab1 *and *Fyn *isoforms

**Figure 1:**
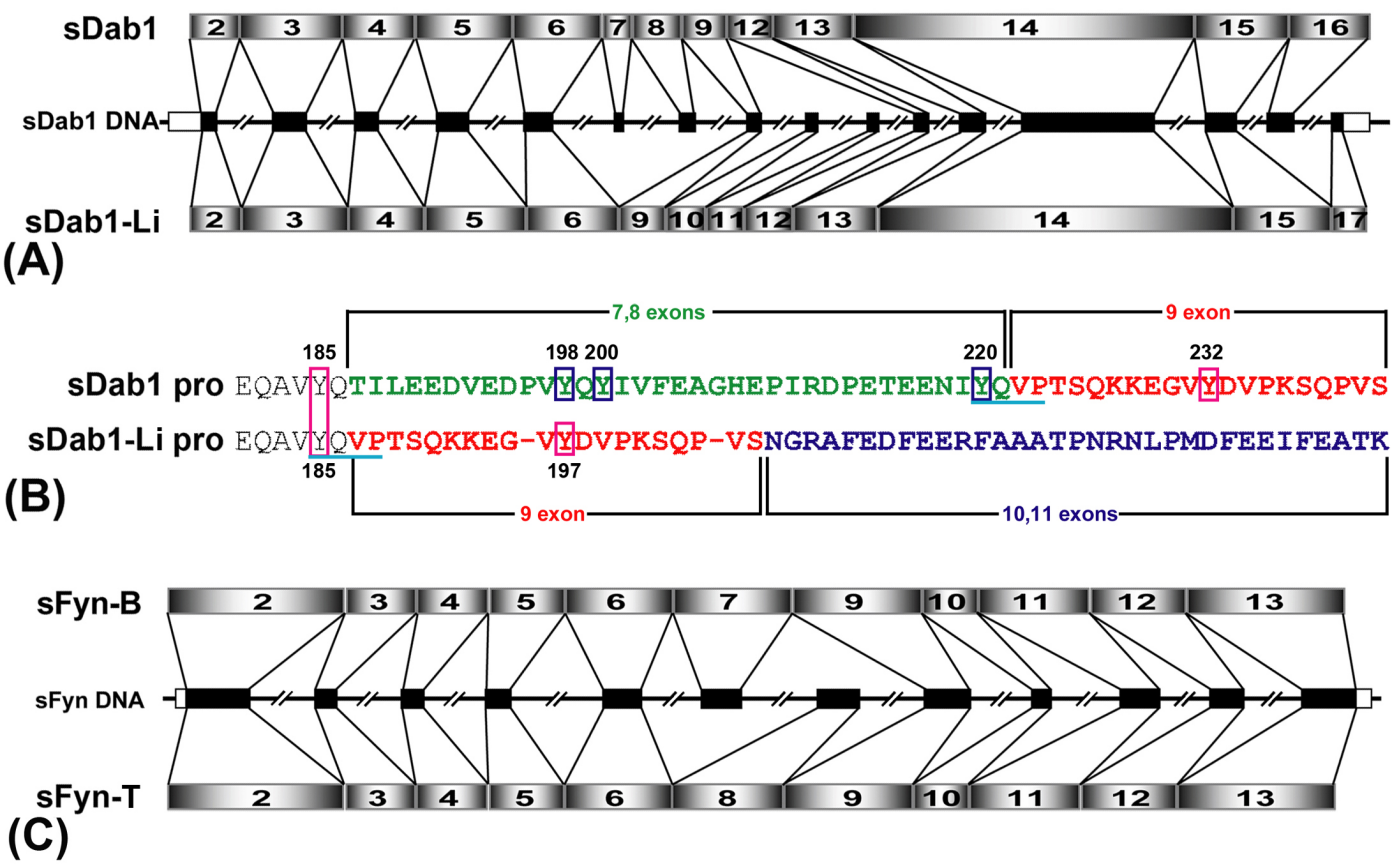
**Organization of the porcine *Dab1 *and *Fyn *genes**. A: Comparison of alternatively spliced isoforms of *sDab1*. The middle row shows genomic organization of the coding region of porcine *Dab1 *gene, which includes the relative positions of all the putative exons (black boxes) and introns (blocked lines). Note that the actual lengths of these exons may slightly vary since the 5'-UTR or 3'-UTR acquired in our study was not complete and the lengths of introns are not respected in this schematic figure. B: Changes of tyrosines after alternative splicing of sDab1. C: Comparison of alternatively spliced isoforms of *sFyn*. The middle row shows the genomic organization of the coding region of porcine *Fyn *gene, which includes the relative positions of all the putative exons (black boxes) and introns (blocked lines).

Using RT-PCR, two isoforms of *Dab1 *were amplified from pig; one was from cerebral cortex cDNA and named *Sus scrofa Disabled 1 *(*sDab1*). This isoform corresponded to the full-length Dab1 protein. The other, shorter isoform was isolated from liver cDNA and named *Sus scrofa Disabled 1-liver *(*sDab1-Li*). The ways to generate both alternatively spliced sDab1 isoforms are shown in Figure 1-A. In the middle row, we show the genomic organization of porcine *Dab1 *gene, which includes relative positions of all the putative exons and introns based on human *Dab1 *genomic structure. Obtained partial mRNAs (including the entire coding regions), consisting of 1867 bp (coding region of *sDab1*: 1668 bp) and 1750 bp (coding region of *sDab1-Li*: 1611), respectively, were verified by sequencing and deposited at Genbank under accession numbers DQ836054 (*sDab1*) and EF444935 (*sDab1-Li*). The sDab1 coding sequence had a high level of similarity to the human (95.6%), mouse (92.0%), rat (92.4%) and chicken (84.3%) nucleotide sequences. The start codons of *sDab1 *and *sDab1-Li *are at the same position in deduced exon 2. Furthermore, *sDab1 *and *sDab1-Li *have two major differences: (i) exon 10 and 11 (corresponding to murine/human exon 9b and exon 9c, respectively[16]) were spliced out in *sDab1*, whereas, exons 7 and 8 were spliced out in *sDab1-Li*; (ii) the stop codon of *sDab1 *was located at exon 16, however, exon 16 was excluded in *sDab1-Li *and it stopped at exon 17. Tyrosine phosphorylation is critical for Dab1 function[2,10]. The deduced protein region corresponding to exon 7 and 8 overlaps with two non-receptor tyrosine kinase recognition sites: Y^198^QY^200^I and Y^220^QVP (Figure 1-B), which means that three functional tyrosine (Y^198^, Y^200 ^and Y^220^) residues were spliced out in *sDab1-Li*.

Using RT-PCR, two isoforms of *Fyn *were amplified from pig, one was from cerebral cortex cDNA which is the porcine counterpart of *Fyn-B *in other species and thus named *Sus scrofa Fyn-brain *(*sFyn-B*); another was from spleen cDNA which is the porcine counterpart of *Fyn-T *in other species and thus was named *Sus scrofa Fyn-T *(*sFyn-T*). The ways to generate both alternatively spliced *sFyn *isoforms are shown in Figure 1-C. In the middle row, we show the genomic organization of porcine *Fyn *gene, which includes the relative positions of all the putative exons and introns based on human Fyn genomic structure. Both isoforms have the same start and stop codon positions in deduced exon 2 and exon 13, respectively. The obtained coding regions, consisting of 1614 bp and 1605 bp, respectively, were verified by sequencing and deposited at Genbank under accession numbers DQ836055 (*sFyn-B*) and FJ195639 (*sFyn-T*). The coding sequence of porcine Fyn-B shares 93.6%, 93.2%, 91.4% and 91.1% identity with its homologues in human, monkey, mouse and rat, respectively. The major difference between *sFyn-B *and *sFyn-T *is that the exon 8 is spliced out in *sFyn-B*, whereas in *sFyn-T *the exon 7 is excluded.

The chromosomal localizations of porcine *Dab1 *and *Fyn *were determined using a porcine radiation hybrid (IMpRH) panel. Porcine *Dab1 *was mapped to chromosome 6q31-35 at a distance of 43cR, 59cR and 72cR to its three closest linked markers SW322 (LOD = 9.51), SW1069 (LOD = 6.59) and SW1680 (LOD = 5.08), respectively. Porcine *Fyn *was mapped to chromosome 1p13 at a distance of 49cR and 70cR to its two closest linked markers SW301 (LOD = 8.59) and SW781 (LOD = 5.35), respectively, confirming and extending data from the NCBI pig genome resource (http://www.ncbi.nlm.nih.gov/genome/guide/pig/).

### Tissue distribution of porcine *Dab1/Dab1-Li *and *Fyn-B/Fyn-T*

**Figure 2:**
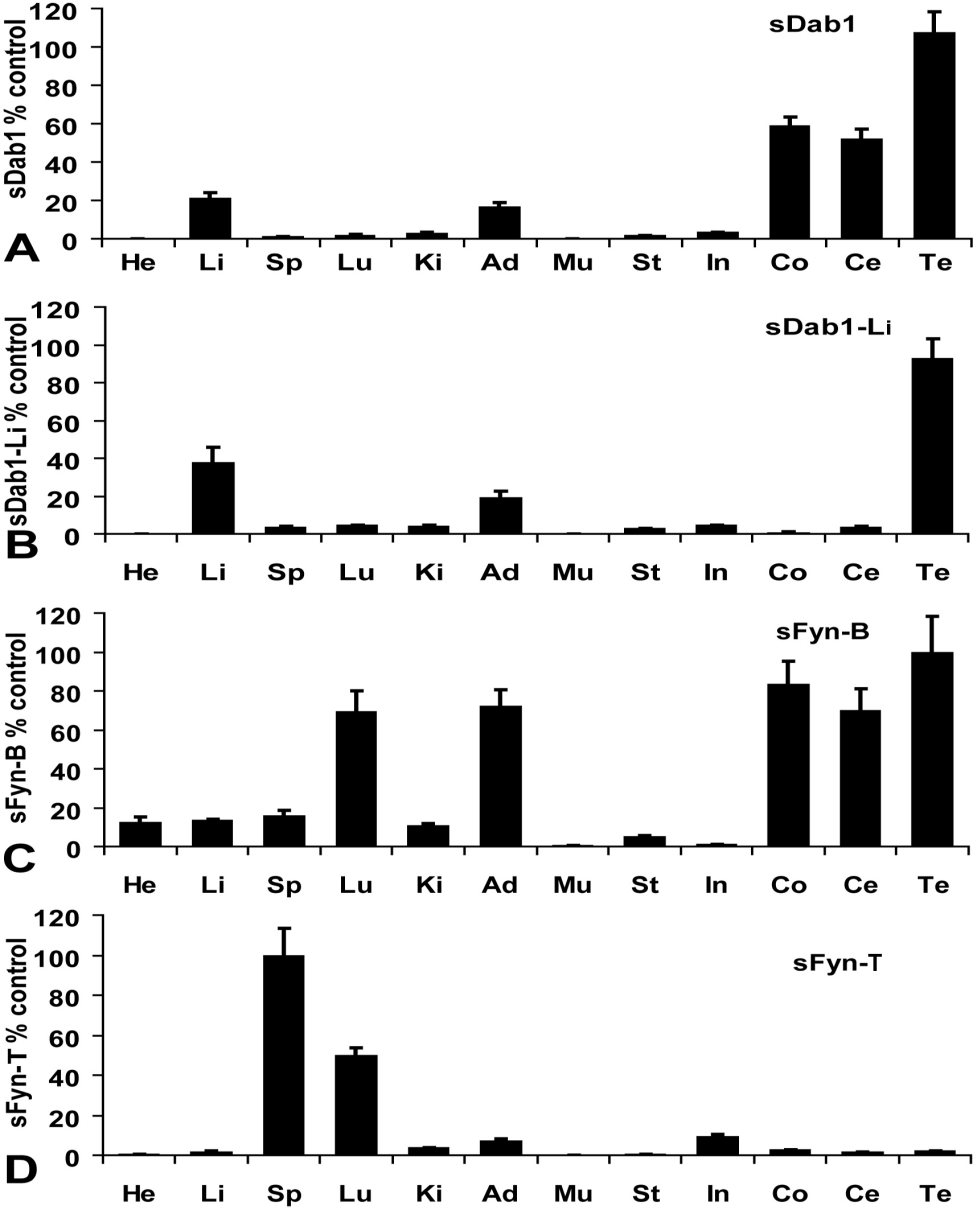
**Expression of *sDab1 *and *sFyn *isoforms (A: *sDab1*, B: *sDab1-Li*, C: *sFyn-B*, D: *sFyn-T*) in various pig tissues**. Total RNA was extracted from 12 different tissues (He: heart; Li: liver; Sp: spleen; Lu: lung; Ki: kidney; Ad: adipose; Mu: muscle; St: stomach; In: intestine; Co: cerebral cortex; Ce: cerebellum; Te: testicle) and subjected to real-time RT-PCR. The expression of *sDab1 *and *sFyn *was calculated as a percentage of *GAPDH *mRNA level in parallel. Data are means ± SEM of four independent experiments.

Real-time quantitative RT-PCR was used to investigate the expression pattern of porcine *Dab1/Dab1-Li *and *Fyn-B/Fyn-T *in various tissues of 2-month-old pigs. Primers 9/10 were designed within *sDab1 *specific exons 7 and 8, while primers 11/12 were designed within *sDab1-Li *specific exons 10 and 11. The forward primer 13 was designed within exon 7, while primer 15 was designed within exon 8, and they both were used together with reverse primer 14 which was designed within exon 9 to detect *sFyn-B *and *sFyn-T*, respectively. As shown in Figure 2-A, B, *sDab1 *and *sDab1-Li *exhibited distinct tissue-specific expression patterns. Full-length *sDab1 *was expressed at relatively high levels in testicle, cerebrum and cerebellum; besides, it was detected at lower levels in liver and adipose tissue, and at very low levels in the rest of the tissues (Figure 2-A). *sDab1-Li *was expressed at relatively high levels in testicle and liver, at lower levels in adipose tissue and very low levels in the rest of the tissues (Figure 2-B). As shown in Figure 2-C, D, *sFyn-B *and s*Fyn-T *also exhibited distinct tissue-specific expression patterns. Compared to *sFyn-B *mRNA, which was abundant in testicle, cerebrum, cerebellum, lung and adipose tissue, *sFyn-T *mRNA was expressed at relatively high levels in porcine spleen and lung. s*Fyn-B *mRNA was expressed at low levels in heart, liver, spleen and kidney; and at very low levels in stomach, muscle and intestine (Figure 2-C), whereas *sFyn-T *was expressed at very low levels except for spleen and lung (Figure 2-D).

### Spatio-temporal expression of porcine *sDab1 *and *sFyn-B*

**Figure 3:**
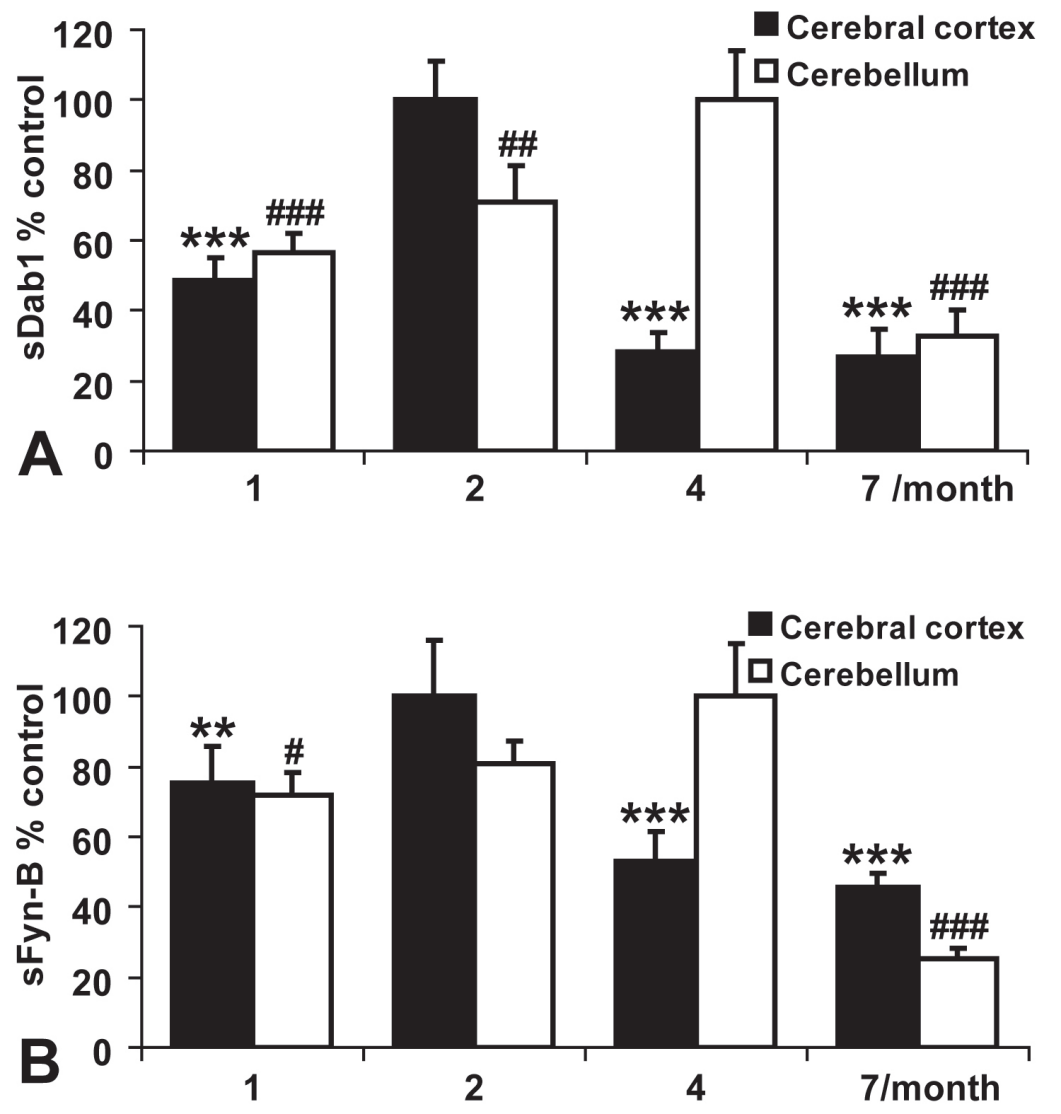
**Expression of *sDab1 *(A) and *sFyn-B *(B) in porcine cerebral cortex and cerebellum at different developmental stages**. Quantitative real-time RT-PCR was performed on mRNAs prepared from cerebral cortex and cerebellum of 1-, 2-, 4- and 7-month-old pigs. The expression of *sDab1 *and *sFyn-B *was calculated as a percentage of *GAPDH *mRNA level in parallel, differences in groups were relative to the highest expression phase (set as 100%) in each group (*p < 0.05, **p < 0.01, ***p < 0.005; ^#^*p *< 0.05, ^##^*p *< 0.01, ^###^*p *< 0.005).

Apart from regulation of neuronal migration during embryonic development, Reelin-Dab1-SFKs signaling is also relevant for the modulation of synaptic plasticity and learning in the adult mouse brain[27,28]. Hence, it is important to test temporal expression patterns of *sDab1 *and *sFyn-B *in the postnatal pig brain. Cerebral cortex and cerebellum of 1-, 2-, 4-, 7-month-old Meishan pigs were chosen to investigate the temporal expression profiles of *sDab1 *and s*Fyn-B*. Of note, they exhibited similar expression profiles. The expression of *sDab1 *and *sFyn-B *reached the highest level in the cerebral cortex at the age of 2 months (p < 0.005/0.01) (Figure 3-A, B; black columns) and at the age of 4 months (p < 0.01/0.05) in the cerebellum (Figure 3-A, B; white columns), respectively, and declined afterwards.

### sDab1 and sDab1-Li are both suggested to be nucleocytoplasmic shuttling proteins

**Figure 4:**
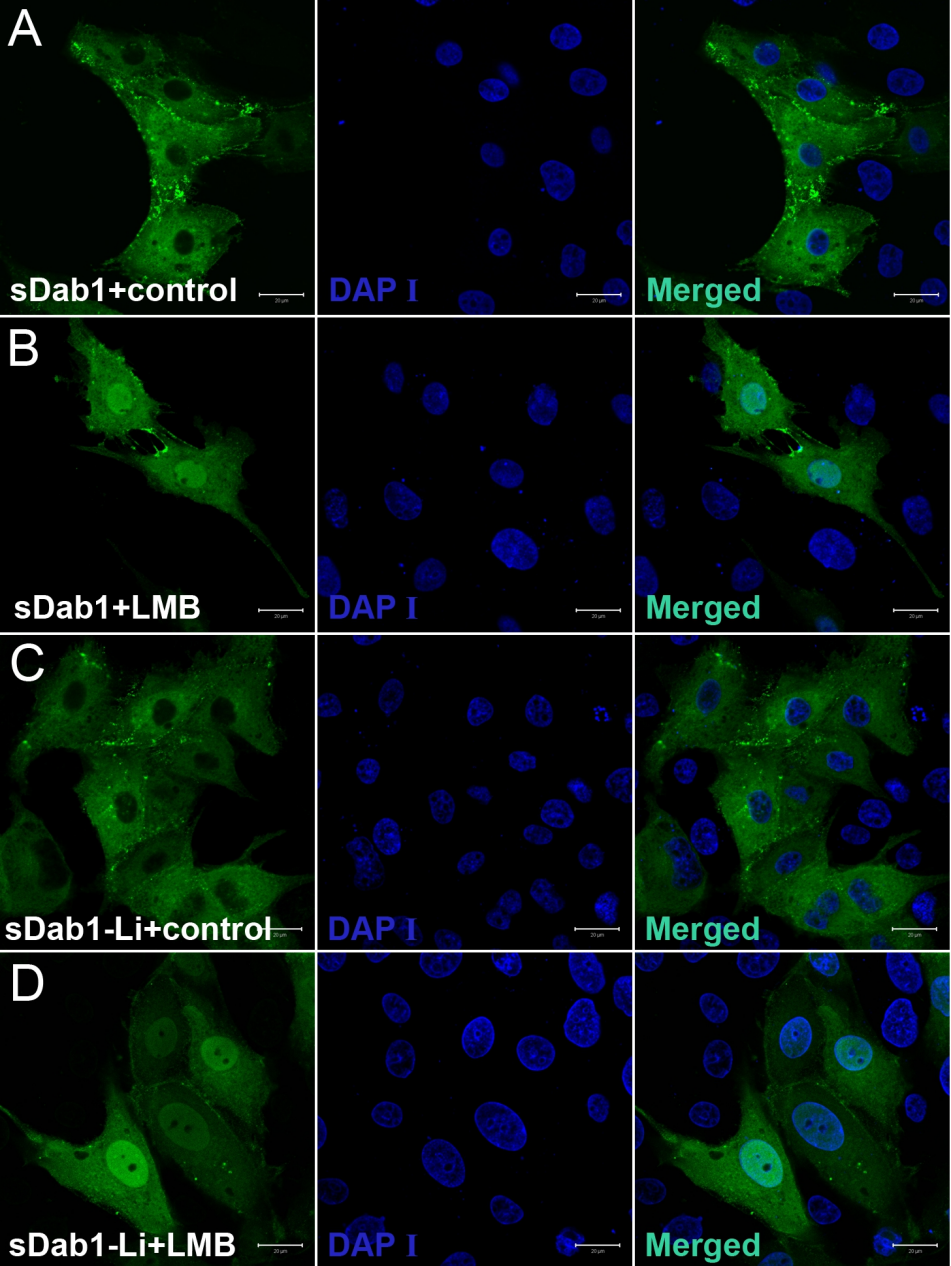
**Nucleocytoplasmic shuttling of sDab1 isoforms in IBRS2 cells**. sDab1-GFP or sDab1-Li-GFP was transiently expressed in IBRS2 cells, and 24 hours after transfection, the cells were treated with methanol (vehicle control) (A or C) or 20 ng/ml LMB (B or D), and after 12 hours of treatment, the cells were fixed and counterstained with DAPI to visualize the nucleus. Scale bars: 20 μm.

It was reported that Dab1 is not only a cytoplasmic adaptor protein, but also a nucleocytoplasmic shuttling protein. Dab1 contains two leucine-rich NES sequences (152LDLRDLFQL160; 462FDISQLNL469) and a bipartite NLS sequence (20RKKGQDRSEATLIKRFK36)[5]. Scanning of the deduced protein sequences of sDab1 and sDab1-Li revealed the presence of these three signal sequences in both porcine Dab1 proteins. To test if both isoforms can shuttle between the nucleus and the cytoplasm, we transiently introduced the sDab1-GFP and sDab1-Li-GFP expression plasmids into IBRS2 cells. 24 h after transfection, the cells were treated with 20 ng/ml LMB (or an equivalent volume of methanol as a vehicle control), a RanGTP-dependent inhibitor of the nuclear export factor exportin 1 (CRM1), for 12 hours. In the absence of LMB, almost 100% of the sDab1- and sDab1-Li-transfected cells showed cytoplasmic distribution. However, LMB treatment led to equal distribution between the cytoplasm and nucleus (Figure 4). This suggests that sDab1 and sDab1-Li are indeed nucleocytoplasmic shuttling proteins and that alternative splicing does not influence their nucleocytoplasmic shuttling properties. The aggregate-like structures seen in Figure 4-A might correspond to filopodia, which have been described in COS-7 cells overexpressing murine Dab1[29].

### Co-localization of sFyn-B with sDab1 and sDab1-Li in primary neurons

**Figure 5:**
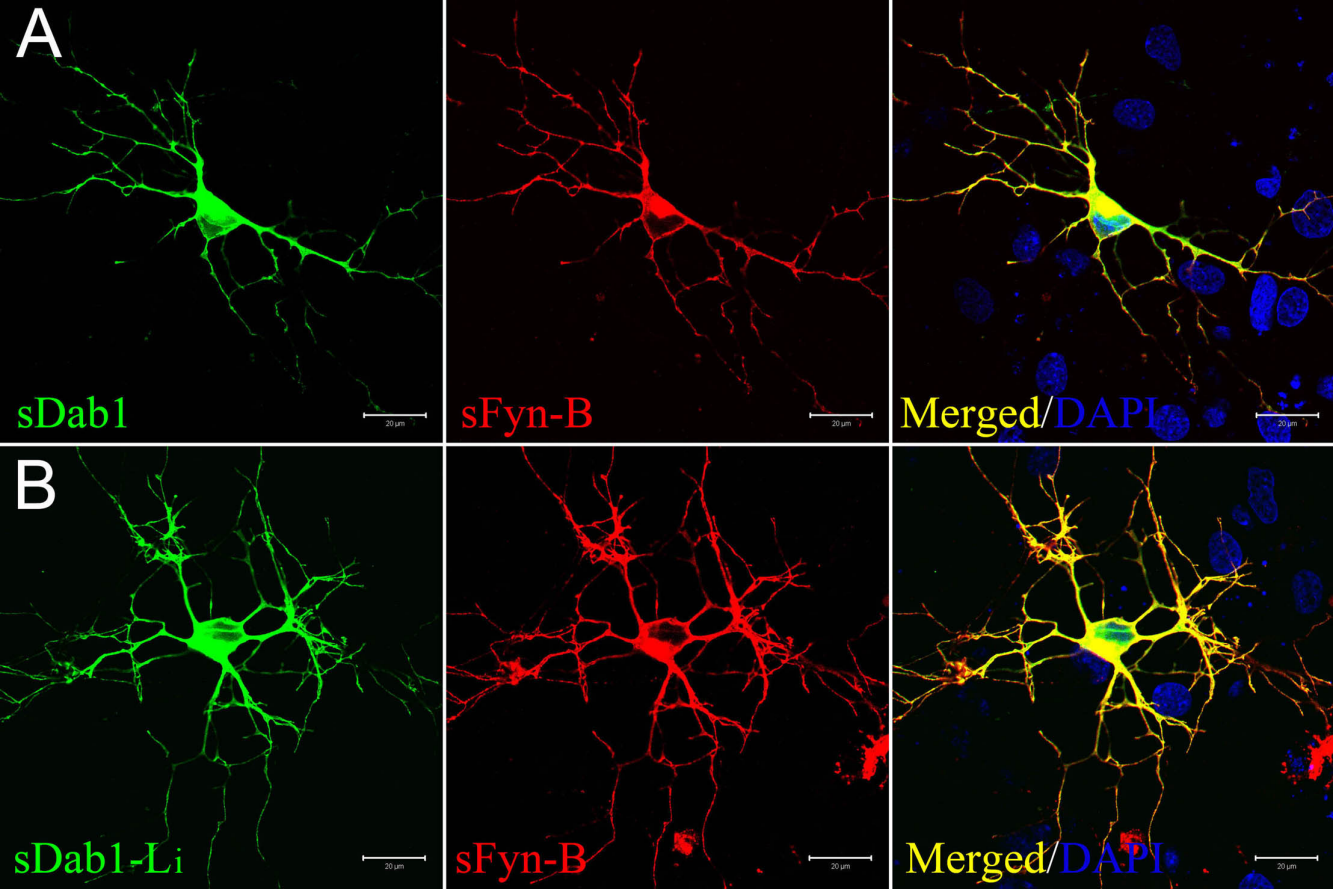
**Co-localization of sDab1 isoforms and sFyn-B in transfected primary neurons**. A: sDab1 -GFP and sFyn-B-DsRed were co-transfected into DIV6 primary hippocampal neurons. B: sDab1-Li-GFP and sFyn-B-DsRed were co-transfected into DIV6 primary hippocampal neurons. 24 hours after transfection, the cells were fixed and counterstained with DAPI (blue) for nuclei. Scale bars: 20 μm.

Reelin activates Fyn to phosphorylate and downregulate Dab1 during brain development[7]. To understand whether sFyn could interact with sDab1 isoforms, we detected their localization while simultaneously overexpressing both of them in primary hippocampal neurons. sFyn-B-Red and sDab1-GFP or sDab1-Li-GFP were co-transfected to DIV6 primary hippocampal neurons. As shown in Figure 5, sFyn-B-Red is co-localized with sDab1-GFP (Figure 5-A), and sFyn-B-Red is co-localized with sDab1-Li-GFP (Figure 5-B), demonstrating that sFyn-B can co-localize with both sDab1 isoforms. These findings indicate that sFyn might interact not only with sDab1, but also with sDab1-Li.

### Phosphorylation of sDab1 isoforms by sFyn at specific sites

**Figure 6:**
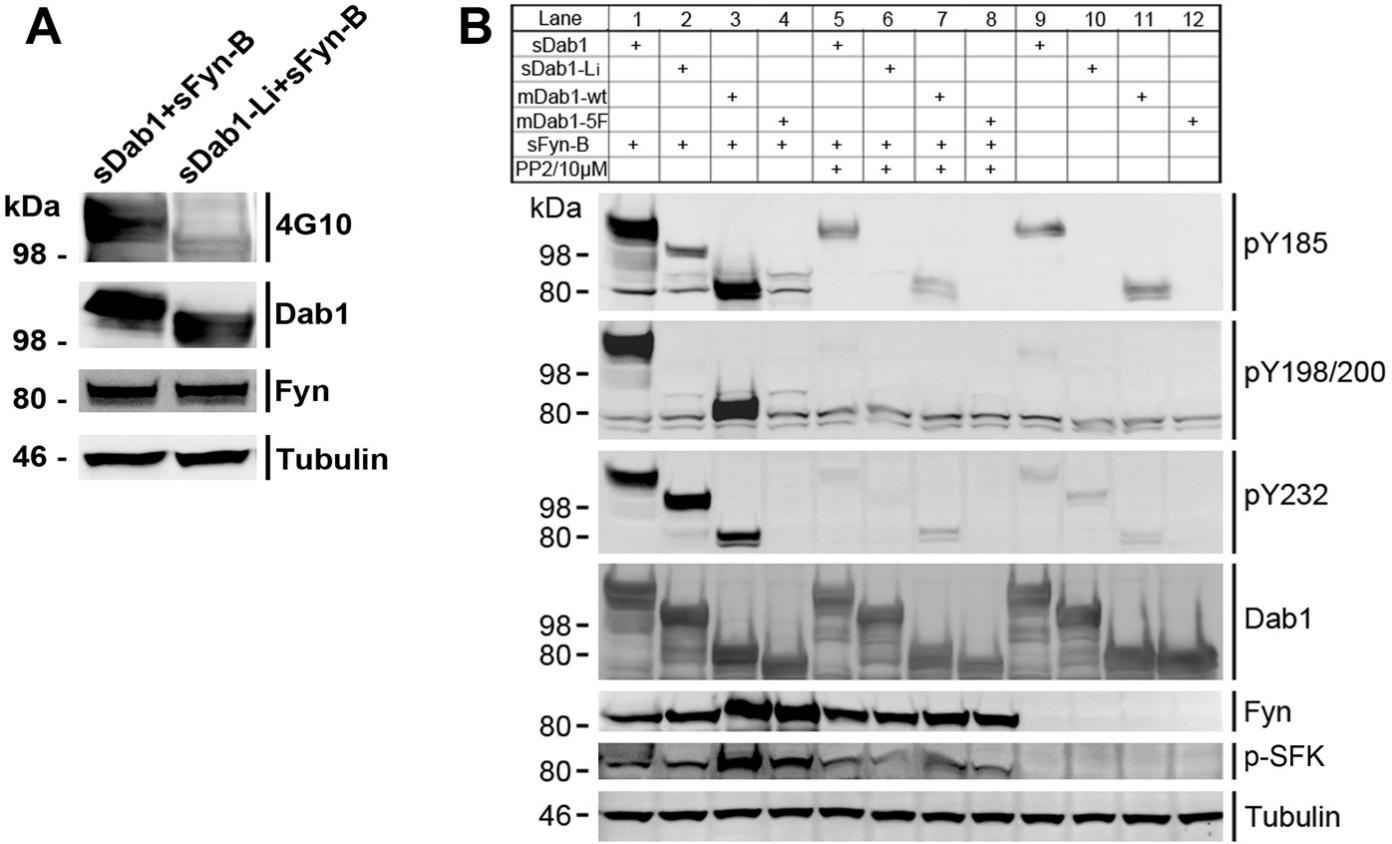
**Phosphorylation of sDab1 and sDab1-Li by sFyn-B in transfected Cos1 cells in vitro**. A: sDab1-GFP or sDab1-Li-GFP was transfected to Cos1 cells along with sFyn-B-GFP. 24 hours after transfection, cells were lysed and analyzed by western blotting using 4G10, anti-Dab1, anti-Fyn and anti-Tubulin. B: Lanes 1-4: sDab1-GFP, sDab1-Li-GFP, mDab1-wt (murine wild-type Dab1 as a positive control) or mDab1-5F (murine mutant form of Dab1 carrying phenylalanine substitutions at residues Y185F, Y198F, Y200F, Y220F and Y232F as a negative control) were expressed in Cos1 cells along with porcine wild-type Fyn-B. Lanes 5-8: PP2, a specific inhibitor of SFKs, was added to the same set of co-transfections as in lanes 1-4. Lanes 9-12: sDab1, sDab1-Li, mDab1-wt or mDab1-5F was separately transfected to Cos1 cells without any exogenous Fyn. 24 hours after transfection, cells were lysed and analyzed by Western blotting using anti Dab1-pY185, anti Dab1-pY198/200 and anti Dab1-pY232. The membranes were also probed with anti-Dab1, anti-Fyn and anti-Tubulin antibodies to confirm protein loading. At least three blots were analyzed for these experiments.

To figure out whether both sDab1 isoforms could be phosphorylated by sFyn in vitro, we co-expressed sFyn-B with either sDab1 or sDab1-Li into Cos1 cells. Then anti-phosphotyrosine antibody 4G10 was used to detect the total tyrosyl-phosphorylation of sDab1 isoforms. The membranes were also probed with anti-Dab1, anti-Fyn and anti-Tubulin antibodies in order to confirm equal protein expression and loading. The result is shown in Figure 6-A, indicating that both sDab1 (full-length form) and sDab1-Li (corresponding to chicken Dab1-E) can indeed be tyrosine-phosphorylated by sFyn-B, although phosphorylation of sDab1-Li was much weaker than that of the full-length sDab1 isoform.

To further identify specific tyrosyl sites of sDab1 isoforms that could be phosphorylated by sFyn, we co-transfected sFyn-B with either sDab1 or sDab1-Li into Cos1 cells (Figure 6-B, lanes 1-2). At the same time, sFyn-B was co-transfected with murine wild-type Dab1 (mDab1-wt) or murine Dab1-5F (mDab1-5F), which was carrying single phenylalanine substitutions at Tyr185, Tyr198, Tyr200, Tyr220 and Tyr232, as positive and negative controls, respectively (Figure 6-B, lanes 3-4). Second, we added PP2, a specific inhibitor of SFKs, to the same set of co-transfections (lanes 5-8). Third, we transfected sDab1, sDab1-Li, mDab1-wt or mDab1-5F separately without any exogenous Fyn (lanes 9-12). Then anti-pY185, anti-pY198/200, anti-pY220 and anti-pY232 antibodies were used to detect tyrosyl-phosphorylation of the respective Dab1 residues by western blotting. The membranes were also probed with anti-Dab1, anti-Fyn and anti-Tubulin antibodies in order to confirm equal protein expression and loading.

As shown in Figure 6-B, anti-pY185 immunoreactivity was detected in lysates of cells cotransfected with sDab1, sDab1-Li or mDab1-wt and sFyn, although the signal of overexpressed sDab1-Li was very weak (lane 3). Anti-pY232 reacted with sDab1, sDab1-Li and mDab1-wt cotransfected with sFyn (lanes 1-3), whereas anti-pY198/200 did not react with sDab1-Li plus sFyn (lane 2). The anti-pY220 antibody was not specific. As one would expect, pretreatment of the cotransfected cells with the SFK inhibitor PP2 (lanes 5-8) or omission of exogenous Fyn (lane 9-12) mitigated or abrogated the tyrosyl phosphorylation of Dab1 isoforms.

Our results suggest that Fyn can phosphorylate the full-length brain isoform of sDab1 at all tyrosyl phosphorylation sites, whereas sDab1-Li is phosphorylated at Y197 (corresponding to Y232 in full-length sDab1) and weakly phosphorylated at Y185 when co-transfected with Fyn in a heterologous overexpression system.

## Discussion

The genomic organization of the Dab1 gene in human and in mouse shows unusual complexity, spreading more than 1100 kb of genomic DNA and being composed of 14 exons encoding the major protein form, some alternatively spliced internal exons, and multiple 5'-exons[16]. Similarly, in zebrafish, the Dab1 gene spans more than 600 kb for a coding region of at least 1.8 kb. By using the obtained coding sequences of Dab1 and Fyn isoforms to search for their correlated genome sequences in the "High Throughput Genomic Sequences" (HTGS) database with megaBLAST, we were able to predict the genomic organization of the porcine Dab1 and Fyn genes. From start codon to stop codon, the porcine Dab1 gene spans more than 150 kb, while the porcine Fyn gene spans more than 52 kb, and they both consist of multiple exons and introns (Table 1, Figure 1). Therefore, our data suggest that the porcine Dab1 gene consists of at least 17 exons (6 alternative exons) and 16 introns, while the porcine Fyn gene consists of at least 13 exons (2 alternative exons) and 12 introns. In addition, almost all junctions between the introns and exons conform to the GT-AG rule (Table 1). Using PCR on pig cerebrum cDNA and alignment of genomic and EST sequences, we were unable to identify exons corresponding to mouse Dab1 forms 217 and 271 in pig. The alternatively spliced pattern of Dab1 is species-specific, but different alternative splicing events give rise to isoforms with different numbers of tyrosines. sDab1-Li is highly similar to chDab1-E, an isoform that has been identified and characterized in chicken retina[18]. The alternatively spliced pattern of Fyn is similar between species. The spliced seventh exon may have been derived from a recombinational event with another gene[30].

**Table 1:** Size and boundary nucleotide sequences of exons and introns of the porcine *Dab1 *and *Fyn *genes

*Dab1*				
**Exon number**	**Exon (nt)**	**5' junction**	**Intron (nt)**	**3' junction**

2 (from start codon)	67	AGAAAG...	?	...agGTCAGG
3	140	CTCAAGgt...	7978	...agGGCGTT
4	99	ACAGGGgt...	67718	...agGCTCTT
5	132	CAGGCGgt...	583	...agGCTGAA
6	120	TATCAGgt...	456	...agACAATT
7 (exists in sDab1)	39	TACCAGgt...	1561	...agTACATT
8 (exists in sDab1)	66	TATCAGgt...	7729	...agGTTCCC
9	60	GTAAGTgt...	28324	...agAACGGC
10 (exists in sDab1-Li)	51	ACGCCGgt...	92	...agAACAGA
11 (exists in sDab1-Li)	48	ACAAAGgt...	4910	...agGCTGTG
12	63	CCCCCCgt...	2345	...agACTCCT
13	109	CCTCAGgt...	8585	...agGTTATG
14	549	ACTCACgt...	4646	...agCTCCCA
15	128	GAAGCCgt...	347	...agCCTGAT
16 (stop codon of sDab1)	111	GTCTGGgt...	13941	...agGAGCTG
17 (stop codon of sDab1-Li)	45			

***Fyn***				

Exon number	Exon (nt)	5' junction	Intron (nt)	3' junction
2 (from start codon)	247	GAACAGgt...	3850	...agGAGTGA
3	97	CAGCTCgt...	7851	...agGGAAGG
4	99	AGAAGAgt...	3193	...agGTGGTA
5	104	CCAAAGgt...	976	...agGTGCCT
6	150	ACTCAGgt...	3179	...tcAGAGAG
7 (exists in sFyn-B)	165	GGATGGgt...	527	...agAGAAAG
8 (exists in sFyn-T)	156	GGCTTGgt...	3094	...agGTACCT
9	180	ATAAAGgt...	1547	...agGAAGCT
10	77	GCACAGgt...	90	...agGTTGCT
11	154	GACAAGgt...	19971	...agGTGCAA
12	133	ACCCAGgt...	10569	...agGCATGA
13 (till stop codon)	209			

Note: "?" means intron was not found by megaBLAST.

Scanning the deduced proteins of sDab1 and sDab1-Li against Prosite (http://www.expasy.org/tools/scanprosite/) indicates that they both contain a PTB/protein interaction (PI) domain from amino acid 37 to 169. These data suggest that both isoforms could compete for the same receptors, but only the isoform that carries target tyrosines can interact with corresponding molecules and transduce the signal, while the other one might work as a dominant negative form. A form of chicken Dab1 that is very similar to sDab1-Li, called chDab1-E, has been shown not to be tyrosine phosphorylated upon Reelin stimulation and not to interact with CrkL[31,32]. The scan of the deduced proteins of sFyn-B and sFyn-T against Prosite suggests that the alternatively spliced sixth exon couldn't change the domain arrangement of Fyn; they share the same domain arrangement, possessing a Src homology 3 (SH3) domain which directs specific association with proline rich motifs, followed by a Src homology 2 (SH2) domain which provides interaction with phosphotyrosine motifs, and a kinase domain responsible for the enzymatic activity.

We mapped porcine *Dab1 *and *Fyn *to chromosome 6q31-35 and 1p13, respectively. The syntenic regions of porcine 6q31-35 and 1p13 on human chromosome are 1p32-31 and 6q21, respectively, where human *Dab1 *[Genbank accession no. NM_021080] and *Fyn *[Genbank accession no. NM_002037] are located, which illustrates the accuracy of our localization. We also mapped porcine *ApoER2 *to chromosome 6q31-35 (unpublished data), which underlines the close functional relationship of porcine Dab1 and ApoER2.

Our results indicate that different isoforms of porcine *Dab1 *and *Fyn *show distinct tissue expression patterns. The high expression levels of *sDab1 *and *sFyn-B *in cerebrum and cerebellum imply their important role in the central nervous system of the pig, as shown for other species. Both *sDab1 *isoforms and *sFyn-B *exhibited the highest expression level in testicle. Besides, porcine *ApoER2 *and *VLDLR *were highly expressed in testicle (unpublished data). It was reported that *Fyn *is required for spermatogenesis[33], therefore, their interaction might exert important functions in the reproductive system. Also, both *sDab1 *isoforms and *sFyn-B *were expressed in adipose tissue, and it was shown that the loss of *Fyn *markedly improved insulin sensitivity[34], hence, they might interact to influence fatty acid metabolism. The high expression level of *sFyn-T *in spleen and lung suggests a role in the immune system, as shown in other species; for instance, Fyn is required for the activation of T cells[35]. Further investigation is needed to explore the multiple roles of sDab1 isoforms in various tissues in addition to the regulation of neuronal migration and neurotransmission.

Our results also suggest that both sDab1 and sDab1-Li can function as nucleocytoplasmic shuttling proteins, and alternative splicing does not influence their nucleocytoplasmic shuttling properties. After inhibition of nuclear export with leptomycin B, sDab1 and sDab1-Li were distributed equally between nucleus and the cytoplasm instead of only being localized in nucleus. One possible reason, besides the fact that sDab1 was overexpressed, could be that the sDab1 isoforms are partly retained by cytoplasmic protein complexes, which prevented them from shuttling. Nucleocytoplasmic shuttling of sDab1 isoforms implies nuclear functions, e.g. participation in transcriptional regulation.

The maturation of postnatal pig brain is comparable to that of humans with respect to structure and development, which makes pig a useful model for studying traumatic injury and nutrition of the developing brain[36]. The porcine brain experiences ongoing developmental processes after birth for several months like human's while mouse or rat brain develops mostly in the prenatal phase[37]. In postnatal pigs, the neocortical synaptophysin expression increases significantly between 2 and 4 months and slightly decreases by 6 months, indicating that porcine neocortex does not reach functional maturity before the age of about 6 months[23]. Pond et al. demonstrated that the development of porcine cerebrum and cerebellum is not synchronized, the growth of cerebellum lags behind cerebrum[38], which is correlated with our observation that the expression of *sDab1 *and *sFyn-B *simultaneously reaches highest level in cerebral cortex at the age of 2 months (Figure 3-A, B) and in cerebellum at the age of 4 months (Figure 3-A, B). These results are similar to the spatio-temporal expression profile of porcine *Cdk5 *that we previously identified[39], suggesting that sDab1 and sFyn-B might play a crucial role in pig brain development, considering that the developmental peak of pig brain is from 2 to 4 months after birth.

Different alternatively spliced patterns of *Dab1 *in different species all generate isoforms with different numbers of phosphorylatable tyrosines. Hence, the alternative splicing of the *Dab1 *gene might be a conserved strategy to achieve functional regulation of its phosphorylation through evolution. Recently, it has been shown that Dab1 can act both as a kinase switch and a scaffold for assembling signaling complexes in vivo through the phosphorylation of the a/b (YQXI, with Tyr185, a; and Tyr198, b) and c/d (YXVP, with Tyr220, c; and Tyr232, d) sites, respectively[12]. The phosphorylation at a/b sites (Tyr185 and Tyr198) of Dab1 stimulates Akt/PI3K signaling and targets phosphorylated Dab1 for degradation[12]. Here, by using anti-phospho-specific-sites Dab1 antibodies, we demonstrated that sFyn-B could phosphorylate sDab1 at Tyr185, -198/200, and -232 as shown previously in mouse[2], and phosphorylate sDab1-Li weakly at Tyr 185 and robustly at Tyr 197, which corresponds to Y232 in full-length sDab1 (Figure 6-B). The weak immunoreactivity of anti-pY185 in lysates of cells cotransfected with sDab1-Li and sFyn can be explained by the observation that Y185 changes its sequence from YQXI to YXVP after splicing of exons 7 and 8, corresponding to a switch from an 'a' site to a 'c' site (Figure 1-B). As a consequence of this splicing event, the sequence surrounding tyrosine 197 switches from a 'b' site to a 'd' site, meaning that it corresponds to Y232 in the full-length porcine Dab1 (Figure 1-B). This is consistent with our result that anti-pY232 but not anti-pY198/200 immunoreacts with sDab1-Li cotransfected with sFyn (Figure 6-B). Here, we studied the functional interaction between porcine Dab1 isoforms and Fyn in vitro. Godbout's group recently showed that chicken Dab1-E, which is similar to sDab1-Li, was not phosphorylated in the absence of Y198/200 and Y220 in ED10 retinal lysates or after Reelin treatment of cultured retinal cells[32]. It will be interesting to determine if sDab1-Li can be tyrosine-phosphorylated in response to Reelin treatment.

## Conclusions

Alternative splicing of porcine Dab1 generates a natural *sDab1-Li *isoform that only carries Y185 and Y197 (corresponding to Y232 in sDab1) sites, which both can be phosphorylated by Fyn in vitro. *sDab1-Li *is highly expressed in peripheral organs. Both isoforms are suggested to be nucleocytoplasmic shuttling proteins. sDab1-Li might regulate cellular responses to different cell signals by acting as a dominant negative form against sDab1. Alternatively, it might be involved in Reelin-independent signaling cascades in peripheral organs.

Besides the essential functions of Dab1 and Fyn in brain development, they both play a role in tumorigenesis. It was reported that the human *DAB1 *gene is located within an unstable common fragile site (CFS) region, and its expression level was decreased in many human cancer samples[40], including epigenetic silencing in pancreatic cancer cell lines[41]. In addition, Fyn is particularly upregulated in various cancers, including, e.g., prostate cancer, glioblastoma multiforma, and melanoma[42]. Hence, further investigation of the functions of Dab1 isoforms and Fyn will also be of potential importance in oncology. Our initial characterization of *sFyn *and *sDab1 *isoforms provides a basic framework for respective studies in the pig as a model organism.

## Methods

### Animals, cell lines, antibodies and inhibitors

Meishan pigs (one, two, four, seven months old, n = 3 at each age) were obtained from the Animal Center of Huazhong Agricultural University (Wuhan, China), and slaughtered after fasting 16-18 hours. Twelve tissues, including heart, liver, spleen, lung, kidney, adipose, muscle, cerebral cortex, cerebellum, stomach, intestine, and testicle were freshly collected and immediately frozen in liquid nitrogen for further use. All experimental procedures were approved by Hubei Province Committee on Laboratory Animal Care.

Cos1 cells and IBRS2 cells were grown in Dulbecco modified Eagle medium (DMEM) containing 10% fetal bovine serum (Hyclone). The rabbit polyclonal anti-Dab1 (1:1000) was purchased from Chemicon. The mouse monoclonal antibody to neuronal class III β-tubulin (TUJ1; 1:2000) was purchased from Covance. The rabbit polyclonal anti-Fyn (1:1000) and the monoclonal anti-phosphotyrosine antibody 4G10 (1:3000) were purchased from Upstate Biotechnology. The rabbit-anti-p-SFK (Tyr416, 1:1000) antibody was purchased from Cell Signaling Technology, Germany. Polyclonal antibodies against phosphorylated Dab1 were prepared by immunizing rabbits with peptides Dab1-pY185 (CEQAVpYQTILEED), Dab1-pY198/200 (CEDPVpYQpYIVFEAG), Dab1-pY220 (CETEENIpYQVPTSQK) and Dab1-pY232 (CKKEGVpYDVPKSQP) coupled to keyhole limpet hemocyan.

PP2 (4-Amino-5-(4-chlorophenyl)-7-(t-butyl) pyrazolo [3, 4-d] pyrimidine), an inhibitor of SFKs, was purchased from Merck.

### RNA isolation and reverse transcription

Total RNA was extracted with Trizol (Invitrogen), followed by treatment with RNase-free DNaseI (Takara) according to manufacturer's instructions, and stored at -80°C. Reverse transcription was carried out by using RevertAid First Strand cDNA Synthesis Kit (Fermentas) as described in the protocol. cDNAs were stored at -20°C.

### Gene Cloning

The full-length coding sequence (CDS) of *sDab1 *and *sFyn-B *was obtained from cerebral cortex cDNA, *sDab1-Li *was from liver cDNA and *sFyn-T *was from spleen cDNA of 2-month-old pig. Human cDNA [Genbank accession no. NM_021080 for *Dab1*, NM_002037 for *Fyn*] was used to search for homologous porcine expression sequence tags (EST) from the "ESTs" database (http://www.ncbi.nlm.nih.gov/genome/seq/BlastGen/BlastGen.cgi?taxid = 9823) with BLAST. Based on the resulting porcine ESTs [BP142065 and CF796289], primer pairs P1/P2 (Table 2) were designed to amplify partial mRNA of porcine Dab1 including the complete CDS from cerebral cortex and liver cDNAs, under the following PCR conditions: 94°C for 5 min, followed by 35 cycles of 94°C for 40 s, 59°C for 40 s, 72°C for 2 min, and a final extension step at 72°C for 10 min. Based on the resulting porcine ESTs [EW140707 and EV932175], primer pairs P3/P4 (Table 2) were designed to amplify the complete CDS of porcine Fyn from cerebral cortex and spleen cDNAs, under the following PCR conditions: 94°C for 5 min, followed by 35 cycles of 94°C for 40 s, 58°C for 40 s, 72°C for 90 s, and a final extension step at 72°C for 10 min. The PCR products were cloned into pMD18-T (Takara) for sequencing.

**Table 2:** Primers used in cloning, chromosomal mapping and real-time RT-PCR for porcine *Dab1 *and *Fyn *isoforms

Primers	Application	Size(bp)	Sequence (5' to 3')
P1	Porcine *Dab1 *cloning	1867/1750	5'-ATGTCAACTGAGACAGAACTTCAAGTAGC-3'
P2			5'-TCCCTCTTCCAAGCGAGTTCC-3'
P3	Porcine *Fyn *cloning	1614/1605	5'-ATGGGCTGTGTGCAATGTAAGGA-3'
P4			5'-TTACAGGTTTTCACCGGGTTGATAC-3'
P5	s*Dab1 *mapping	321	5'-ATGACCGACTGGGCTTACTGTTC-3'
P6			5'-CCAAATCACACAGCTTGAAAGAGTC-3'
P7	s*Fyn *mapping	261	5'-CTGCCTCAGATTTCCAGACGC-3'
P8			5'-AGCAGGATGAGGTCAGGAGCC-3'
P9	*sDab1 *expression	207	5'-CCATCCAAATCATCTGCATCCC-3'
P10			5'-GACCTGTGCTATCTAGCTACCGGC-3'
P11	*sDab1-Li *expression	176	5'-GTATCAGGTTCCCACCAGCCAA-3'
P12			5'-GGGTCACAGCCTTTGTTGCCT-3'
P13	*sFyn-B *expression	179	5'-AAAGGGATGCCAAGGCTTACC-3'
P14			5'-GACATTGTGCCTGGTTTGAGAGTC-3'
P15	*sFyn-T *expression	186	5'-AACTGTGATTGCATCGAGTTGTACC-3'
P14			5'-GACATTGTGCCTGGTTTGAGAGTC-3'
P17	*GAPDH *expression	220	5'-GCAAATTCCACGGCACAGTCA-3'
P18			5'-TCAGCAGAAGGGGCAGAGATG-3'

### Chromosomal mapping

The radiation hybrid (IMpRH) panel containing 118 hybrid clones[43] was used for chromosomal localization. Primer pairs (P5/P6 for *Dab1*, P7/P8 for *Fyn*, Table 2) were designed according to pig genomic DNA sequences containing partial regions of *Dab1*and *Fyn *(Trace Archive ti no. **811166877** for *Dab1*, **2212586528** for *Fyn*, http://www.ncbi.nlm.nih.gov/Traces/trace.cgi?cmd=stat&f=xml_list_arrivals_retrievals&m=main&s=main). The PCR conditions were 5 min at 94°C, followed by 33 cycles of 30 s at 94°C, 30 s at 58°C, 30 s at 72°C, and a final extension of 5 min at 72°C. The resulting PCR fragments were analyzed by the IMpRH mapping tool[44] (https://www-lgc.toulouse.inra.fr/internet/). The locations were inferred from the positions of the closest linked markers on the cytogenetic map. The results were considered credible when LOD > 5.0. We have cloned partial DNA sequences of *Dab1 *[321 bp, GenBank accession no. GU585849] and *Fyn *[261 bp, GenBank accession no. EF378595] for chromosomal mapping. The IMpRH procedure for each gene was accompanied by two negative controls (with no template or with hamster genomic DNA as the template) and a positive control (with porcine genomic DNA as the template).

### Real-time quantitative PCR

The tissue distribution of porcine *Dab1 *and *Fyn *isoforms was examined by real-time quantitative RT-PCR (qRT-PCR) in twelve different tissues from 2-month-old Meishan pigs. The spatio-temporal expression of porcine *sDab1 *and *sFyn-B *was carried out in cerebral cortex and cerebellum cDNAs from 1-, 2-, 4- and 7-month-old Meishan pigs. qRT-PCR was performed using a Bio-Rad iQ™5 Real Time PCR Detection System. P9/P10 and P11/P12 primer pairs (Table 2) were designed based on the non-homologous region of coding sequences of *sDab1 *and *sDab1-Li *we isolated, respectively. P13/P14 and P15/P16 primer pairs (Table 2) were designed based on the non-homologous region coding sequences of *sFyn-B *and *sFyn-T*, respectively. The 2 × SYBR^® ^Green Realtime PCR Master Mix (Toyobo) was used for the real-time quantification of PCR products. Samples were amplified in 4 duplicates with the following parameters: 95°C for 2 min, followed by 40 cycles of 95°C for 10 s, 58°C for 15 s and 72°C for 15 s. Melting curve analysis was performed following PCR cycling in order to confirm that fluorescent signal was generated only from specific cDNA transcripts. As an internal control, the housekeeping gene *GAPDH *[Genbank accession no. AF017079] was amplified in each reaction with commensurate template using P17/P18 primer pairs. Relative gene expression was calculated by using the 2^-ΔΔCt ^method[45]. The tissue that exhibited highest expression level was chosen as a reference in each respective reaction.

### Leptomycin B (LMB) treatment

For CRM1-dependent nuclear export inhibition experiments, the cells were treated with LMB (Sigma) at 20 ng/ml or the same volume of methanol as a vehicle control 24 hours after transfection and incubated for 12 hours, then fixed and counterstained with DAPI (1:10,000; Invitrogen). Images were collected using a confocal microscope (LSM 510, Carl Zeiss).

### Primary hippocampal neuron culture

For primary neuron cultures, new-born (P0) wild-type rats (Wistar line; n = 4 for each group) were used. The hippocampus was dissected and put into ice-cold HBSS. After removal of the meninges, the hippocampi of both hemispheres were collected and maintained in 15 ml Falcon tubes (BD Biosciences) containing 3 ml of 0.5% Trypsin and 0.53 mM EDTAx4Na (Invitrogen) and incubated at 37°C for 15 min. The hippocampi were then washed twice in ice-cold HBSS and manually triturated with a polished glass Pasteur pipette for several times. Then, the cells were centrifuged at 800 × g for 5 min. The sediment was collected in fresh Falcon tubes, and cell pellets were resuspended in 1 ml of Neurobasal-A medium (Invitrogen), supplemented with 2% B27 (Invitrogen), 1 mM Glutamax (Invitrogen), 100 U/ml penicillin and 100 μg/ml streptomycin (Invitrogen). Hippocampal cells were counted using a hemocytometer and suspended at a density of 5 × 10^5 ^cells in 0.5 ml aliquots on glass coverslips inserted in 24-well plates. The coverslips had been coated overnight with a solution of 20 μg/ml poly-L-ornithine (Sigma) prepared in borate buffer pH 8.1. After 2 h of incubation in a humidified incubator at 37°C and 5% CO_2 _to allow for cell attachment, the medium was changed to remove dead cells. After 6 days of incubation, cells were used for co-transfection.

### Plasmids construction and transient transfection

The complete coding sequence of *sDab1 *and *sDab1-Li *[Genbank accession number: DQ836054,EF444935] was subcloned into pEGFP-N1 and termed sDab1-GFP and sDab1-Li-GFP; sFyn-B [Genbank accession number: DQ836055] was subcloned into pDsRed-Express-N1 and designated sFyn-B-DsRed. All the constructs were verified by DNA sequencing. Full-length murine Dab1 (wildtype and the 5F mutant carrying the mutations Y185F, Y198F, Y200F, Y220F, and Y232F, respectively) was cut out of the pECE vector[10] with BamHI and ligated into pcDNA3.1 vector. Insertion in the proper orientation was checked by restriction digest. For transient transfection, cells in 24 wells were incubated with a mixture of 1 μg plasmid DNA and Lipofectamin 2000, under the conditions recommended by the manufacturer (Invitrogen). After 24 hours, cells were fixed and counterstained with DAPI.

### Western blotting

After washing three times in cold PBS, the cells were resuspended in ice-cold hypertonic lysis buffer [pH 7.6, 50 mM Tris-HCl, 150 mM NaCl, 5 mM EDTA-Na_2_, 1% (v/v) Nonidet P-40, 1% Triton X-100, 0.5% (w/v) SDS, 0.25% (w/v) sodium deoxycholate] supplemented with 1% protease inhibitor (Sigma) and phosphatase inhibitor cocktails (Sigma). The extracts were centrifuged for 20 min at 12,000 g at 4°C. The resulting crude supernatants were taken, and protein concentration was measured by using BCA Protein Assay Kit (Pierce). Aliquots of the supernatants were immediately used for western blotting or were stored at -80°C until use. Ten to twenty micrograms of each sample were diluted in ultrapure water containing 1 × NuPAGE sample buffer (Invitrogen) and 1 × NuPAGE reducing agent (Invitrogen) to final volumes of 20 μl, boiled at 95°C for 5 min, and then immediately placed on ice. The samples were separated by NuPAGE Bis-Tris, pH 7.0, 4-12% PAGE (Invitrogen) with 1 × NuPAGE MES-SDS running buffer (Invitrogen) and transferred electrophoretically to polyvinylidene fluoride (PVDF) membranes with 1 × NuPAGE transfer buffer (Invitrogen). For blotting, membranes were blocked with 5% (w/v) nonfat milk in Tris-buffered saline (TBS) at RT for 2 h and subsequently incubated with primary antibodies in blocking solution at 4°C overnight. After extensive washing, filters were incubated with a 1:3000 dilution of goat anti-rabbit/mouse HRP-labeled antibody. ECL substrate kit (Amersham, Piscataway, NJ) was used for chemiluminscence detection of the signals with autoradiography film. At least four western blots were analyzed for each experiment.

### Data analysis

The data were represented as mean ± SEM; differences between groups were analyzed by one-way ANOVA followed by Tukey post hoc test, using GraphPad Prism. A P-value of less than 0.05 was considered to be statistically significant.

## Authors' contributions

HL carried out the molecular genetic and cellular studies along with western blotting and qRT-PCR experiments, and participated in conceiving the study. HHB participated in the design of the study, and in the evaluation of the results. TL participated in the design of cloning experiment. XC participated in the western blotting assay. JY participated in the qRT-PCR experiment. JH and HHB provided reagents. JH participated in the evaluation of the results. MF participated in the design of the study and in the evaluation of the results. ZY conceived the study, and participated in its design and coordination. HL and HHB wrote the manuscript. All authors read and approved the final manuscript.
